# Editorial: The role of sex in coronary artery disease

**DOI:** 10.3389/fcvm.2023.1220439

**Published:** 2023-05-22

**Authors:** Hamidreza Goodarzynejad, Mahmood Sheikh Fathollahi, Akbar Shafiee

**Affiliations:** ^1^ Family Medicine Teaching Unit, North York General Hospital, Toronto, Canada; ^2^ Tehran Heart Center, Cardiovascular Diseases Research Institute, Tehran University of Medical Sciences, Tehran, Iran; ^3^ Rajaei Cardiovascular, Medical and Research Center, Iran University of Medical Sciences, Tehran, Iran

**Keywords:** coronary artery disease, gender, sex, ischemic heart disease, coronary atherosclerosis, coronary heart disease, myocardial infarction

**Editorial on the Research Topic** The Role of Sex in Coronary Artery Disease

## Introduction

Although coronary artery disease (CAD) has been primarily considered as a man's disease and for long time women were underrepresented in CAD clinical trials, women, as compared to men, have shown far worse clinical outcomes following CAD-related events (1). Sex constitutes an easily available and cost-free biological variable that frequently acts as a disease modulator; however, investigators and reviewers are often at a loss about the issue of sex differences (2). The present Frontiers Research Topic aimed to analyze the differences between men and women in awareness, risk factors, pathophysiology, prevention, clinical presentation, diagnostic evaluation, and management of CAD. In this regard, an international selection of investigators contributed original data and up to date reviews to increase our current understanding on the role of sex in CAD.

Seven articles included in our special issue that cover the main themes of the topic. Helman et al. offer an overview of sex differences within the mammalian stress response and the interactions between sex, stress and CAD. The authors assume that the biology of stress (and its influences on health and disease) must be viewed as distinct in men and women. They suggest that not only does repetitive or continued stress promote the development of chronic diseases including CAD, acute mental stress also acts as a potent trigger and is strongly associated with cardiovascular events with greater propensity to microvascular dysfunction and ischemia in women. There is evidence that a relation between mental stress with major adverse cardiac events exists only in women but not men (3). Chronic forms of psychosocial stress also appear to have greater effects on women compared to men. For example, a 2- to 4-fold higher risk of stress-dependent myocardial infarction in women (4, 5), and up to 10-fold higher risk of Takotsubo syndrome—a stress-dependent coronary-myocardial disorder most prevalent in post-menopausal women—(6) has been reported. By discussing the multiple mechanisms by which chronic stress can influence pathophysiological aspects of cardiovascular disorders and the effects of sex on them, the authors highlighted how a more detailed exploration into the mechanisms of sex and stress biology is essential to develop targeted approaches to CAD prevention and therapy in men and women separately. They also emphasized that apart from clear sex-related biological differences, stress responses are similarly influenced by external determinants such as socio-cultural and economic factors. Therefore, significant improvements in the prevention of chronic diseases including CAD in women can be made at fundamental socio-economic levels.

Despite receiving growing attention in recent years, health disparities in women presenting with CAD still exist causing worse clinical outcomes in women than in men (7, 8). Higher patient and system delays are still experienced by women and they continue to receive less aggressive drugs and less invasive treatments. Using data analytics of electronic medical records of ∼32,000 patients with CAD treated at the UCSF Medical Center over an 8-year period, Panahiazar et al. studied the gender-based time discrepancy in diagnosis of CAD. Of patients who eventually diagnosed with severe CAD, there was a greater delay in the time between prescribing aspirin and/or any other cardiovascular medications and prescribing beta-blockers—a known drug to reduce cardiovascular risk—or ordering the electrocardiogram test for women. Women, compared to men, also showed a significantly longer interval between their first physician encounter indicative of CAD and their first diagnostic cardiac catheterization, while the time difference from diagnostic Cardiac Catheterization to coronary artery bypass graft (CABG) was not statistically significant. In another article in the collection, using data of female patients assigned to the primary percutaneous coronary intervention (pPCI) arms of three randomized studies, Motovska et al. compared the combined cohort of patients (*n* = 159) enrolled in the PRAGUE-1 (study period: 1997–1999; female/male ratio = 29/72) and the PRAGUE-2 (1999–2002; 130/299) with those of the PRAGUE-18 study (2013–2016; 299/921). The results showed that the risk of death within 30-days after the index event altered significantly from 8.2% in women undergoing pPCI in the PRAGUE-1 and -2 studies to 3.3% in women enrolled to the PRAGUE-18 study. However, the proportion of women with time to reperfusion more than 6 h after the onset of symptoms remained unacceptably high. The authors concluded that progress in the management of ST elevation myocardial infarction (STEMI), including advancements in procedural instrumentation and technique, and adjuvant antiplatelet therapy, have brought about favorable prognoses for women with acute MI. Despite this, delayed diagnosis and medical care-related delays make the outcome worse in a large proportion of women.

Myocardial infarction with non-obstructive coronary arteries (MINOCA) is a syndrome often overlooked. In this topic, Yildiz et al. comprehensively reviewed its prevalence, characteristics, prognosis, diagnosis, and treatment. They discussed that this syndrome typically affects women with a heterogenous working diagnosis that is understudied, underdiagnosed, and undertreated, and that it has bad prognosis similar to patients with MI and obstructive CAD. They provided evidence that a wide variety of underlying pathophysiologic mechanisms can lead to MINOCA and MINOCA-mimickers. Thus, to guide treatment, it is essential to detect the underlying mechanism using diagnostic workup including multimodality advanced imaging. Further researches are required to guide the specific treatment of the underlying mechanism.

Notably, non-obstructive CAD is linked to coronary microvascular dysfunction (CMD) that is more often seen in women compared to men (9). As women with chest pain and non-obstructive CAD have a two-fold increased risk to develop obstructive CAD events in the next 5–8 years (10), the early diagnosis of CMD, by measuring coronary blood flow reserve, and a better understanding of underlying contributors to sex differences in CMD is warranted. In this context, sex differences in contributors to CMD were investigated by Kwan et al., comparing relations between myocardial perfusion reserve index (MPRI), semi-quantitative measure of perfusion validated as a measure of CMD (11), vs. traditional cardiovascular risk factors and markers of cardiac structure/function in sex-stratified populations. The underlying pathophysiology of CMD was intriguingly proposed by the authors to differ between men and women, as MPRI showed an association with T1 times and LVMI in women vs. T1 times and aortic diameter in men. In addition, reductions in MPRI in men was associated with diabetes and hyperlipidemia while it was not associated with any of traditional cardiovascular risk factors in women.

In a meta-analysis of systematic reviews, Leng et al., for the first time, explored the sex-dependent association of periodontal diseases with CVD. It was observed that there is a significant association between periodontal disease and the risk of CVD independent of sex, with a summary odds ratio of 1.22 for women and 1.11 for men. However, sex differences were found in the CAD subgroup analysis where men with periodontal disease in comparison with women, showed a higher risk of CAD. The authors proposed that the sex-specific association between periodontal disease and CVD may be different in various types of CVDs indicating the need for further analysis in the future. Finally, given that heart failure (HF) is a common complication in patients with acute coronary syndrome (ACS), particularly in women, Yan et al. examined the risk predictors for new-onset HF after ACS by developing a simple nomogram to optimize the clinical management for women. They stated that the novel nomogram can help identify female individuals with ACS who are at high risk of developing HF after discharge and facilitate communication between female patients and physicians.

Overall, the present Research Topic expand our knowledge on sex differences in the CAD. Obviously, the articles collected here represent only a small glimpse of the many lines of research that are ongoing on this topic. As recently illustrated in a comprehensive review, sex and gender can both impact on almost every organ and every disease (12). Not only is it essential to include sex or gender as a variable in the design of studies, but also is of importance to perform sex/gender stratified analyses, as these can reveal other influences. In fact, in May 2014, the National Institutes of Health (NIH) announced the intention to “require applicants to consider sex as a biological variable (SABV) in the design and analysis of NIH-funded research involving animals and cells.” (13) To provide more reliable evidence for CAD diagnosis and treatment with the goal of personalized therapeutics in both men and women, more women of all age groups must be included in future randomized controlled trials.
